# Anomalous in-plane anisotropic Raman response of monoclinic semimetal 1 T´-MoTe_**2**_

**DOI:** 10.1038/s41598-017-01874-2

**Published:** 2017-05-11

**Authors:** Qingjun Song, Haifeng Wang, Xingchen Pan, Xiaolong Xu, Yilun Wang, Yanping Li, Fengqi Song, Xiangang Wan, Yu Ye, Lun Dai

**Affiliations:** 1 State Key Lab for Mesoscopic Physics and School of Physics, Peking University, Beijing, 100871 China; 2 Collaborative Innovation Center of Quantum Matter, Beijing, 100871 China; 3 National Laboratory of Solid State Microstructures, College of Physics, Nanjing University, Nanjing, 210093 China; 4 Collaborative Innovation Center of Advanced Microstructures, Nanjing University, Nanjing, 210093 China

## Abstract

The recently discovered two-dimensional (2D) semimetal 1 T´-MoTe_2_ exhibits colossal magnetoresistance and superconductivity, driving a strong research interest in the material’s quantum phenomena. Unlike the typical hexagonal structure found in many 2D materials, the 1 T´-MoTe_2_ lattice has strong in-plane anisotropy. A full understanding of the anisotropy is necessary for the fabrication of future devices which may exploit these quantum and topological properties, yet a detailed study of the material’s anisotropy is currently lacking. While angle resolved Raman spectroscopy has been used to study anisotropic 2D materials, such as black phosphorus, there has been no in-depth study of the Raman dependence of 1 T´-MoTe_2_ on different layer numbers and excitation energies. Here, our angle resolved Raman spectroscopy shows intricate Raman anisotropy dependences of 1 T´-MoTe_2_ on polarization, flake thickness (from single layer to bulk), photon, and phonon energies. Using a Paczek approximation, the anisotropic Raman response can be captured in a classical framework. Quantum mechanically, first-principle calculations and group theory reveal that the anisotropic electron-photon and electron-phonon interactions are nontrivial in the observed responses. This study is a crucial step to enable potential applications of 1 T´-MoTe_2_ in novel electronic and optoelectronic devices where the anisotropic properties might be utilized for increased functionality and performance.

## Introduction

Transition metal dichalcogenides (TMDCs) are two-dimensional (2D) materials that have emerged as appealing material systems due to their variable electrical band gap and strong spin-orbit coupling. In addition, a rich and diverse amount of physical phenomena have been realized, from correlated charge density waves and superconductivities, to device-driven transistors, sensors, spintronics, and valley-optoelectronics^1–5^. Recently, 2D materials with lower symmetry have attracted increased attention, due to the significant in-plane anisotropy in their electrical, optical and thermal properties^6–12^. For example, the reduced symmetry of black phosphorus has resulted in the in-plane anisotropic mobility, absorption, and thermal conductivity^6–8^. Whereas many TMDCs are in the 2 H phase and are semiconducting, monoclinic 1 T´-MoTe_2_ is semi-metallic^13^. By decreasing the temperature, the monoclinic 1 T´ crystal phase changes to the orthorhombic Td phase, resulting in observations of huge magnetoresistance, type-II Weyl semimetal Fermi arcs, and potentially providing a route towards the quantum spin Hall effect^14–21^. Both 2 H and monoclinic 1 T´-MoTe_2_ are stable at room temperature, and the few-layer crystals can be obtained through the chemical vapor deposition method or mechanical exfoliation^22,23^. However, the studies regarding monoclinic 2D materials and their anisotropic properties are still limited, which is a crucially necessary for the future design and realization of devices with emergent quantum and topological properties for enhanced performance.

Raman spectroscopy is a conventional and non-destructive technique for the characterization of the crystal structure^24^. In 2D materials, Raman spectroscopy is a powerful tool that is typically used to determine the number of layers and provide insight into the doping, strain, and crystal phases^25–29^. Thus, the angle resolved polarized Raman response of monoclinic 1 T´-MoTe_2_ can reveal the material’s anisotropic light-matter interaction, necessary for the improved design of electronic, thermoelectric and optoelectronic devices^30^. Here, we study the anisotropic Raman response of monoclinic 1 T´-MoTe_2_ from single layer to bulk using two different measurement methods. By rotating the sample, the symmetries of the detected modes can be identified through their periods of intensity variation. This method allows us to easily and rapidly identify the crystalline orientation of 1 T´-MoTe_2_ using the *A*_g_ (*A*´) modes with maximum Raman intensities. In addition, the anisotropic Raman response’s dependence on the 1 T´-MoTe_2_ thickness and photon excitation wavelength are carried out by rotating the incident light polarization method, while fixing the sample orientation and scattered light polarization. Using this method, the polar plots of the intensities for all detected modes exhibit a two-lobed shape, while the main-axis orientations for different symmetric modes are different. Their polarization-dependent intensities roughly coincide with the semi-classical model based on the Placzek approximation. Using the full quantum model based on the density functional and quantum perturbation theories, we demonstrate that the anisotropy of Raman modes is both influenced by the anisotropic electron-photon interaction and the anisotropic electron-phonon interaction. Our research not only explores the in-plane anisotropic intensities of Raman modes in monoclinic 2D 1 T´-MoTe_2_, but also reveals the physical origin of the anisotropic Raman response, beneficial for the design of future devices which utilize the anisotropic optical, electrical, and mechanical properties of TMDCs^30^.

## Results and Discussions

### Raman Spectra of 1 T´-MoTe_2_

**Figure 1:**
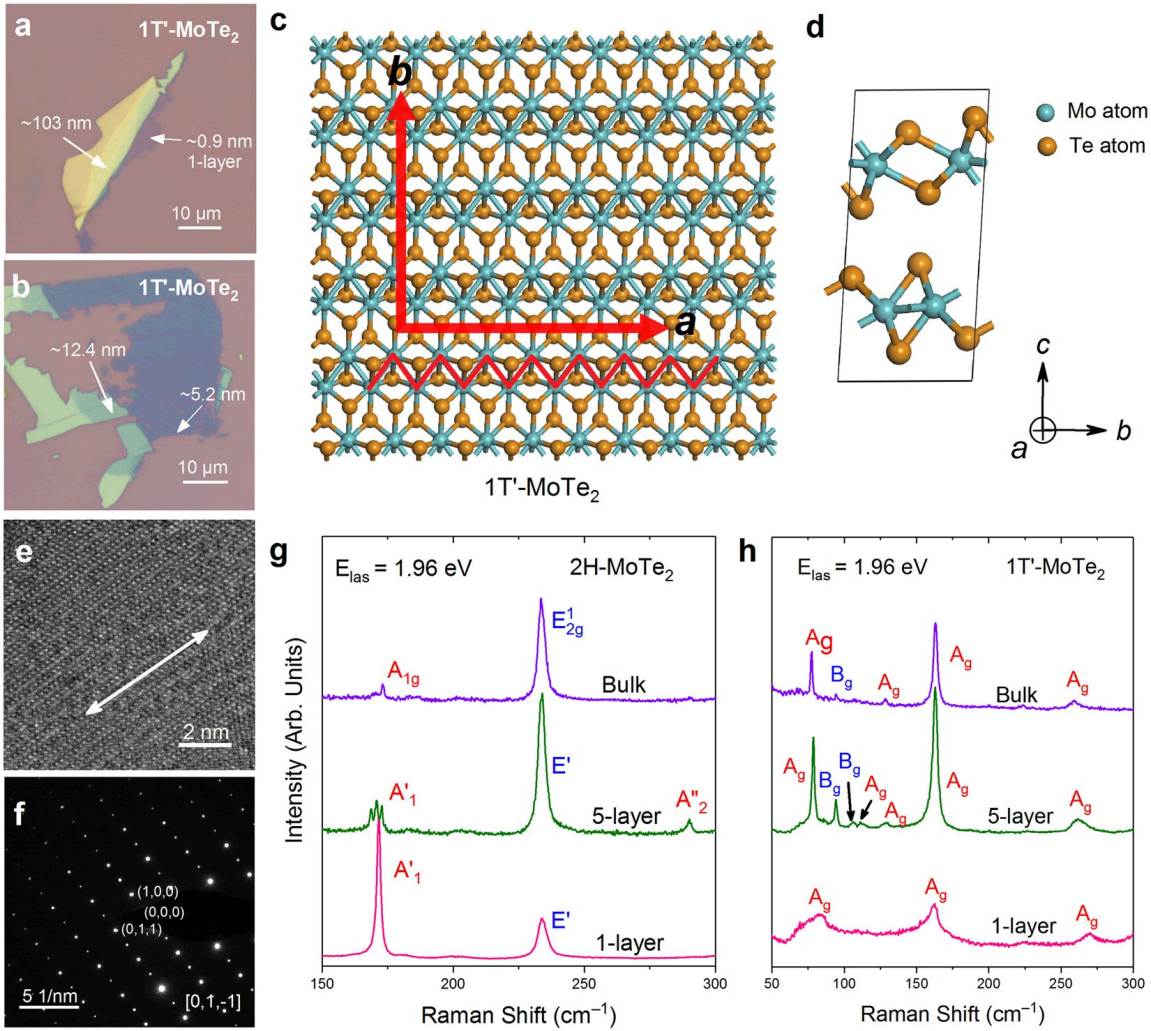
(**a,b**) The optical images of exfoliated single-layer and few-layer 1 T´-MoTe_2_ on a 285 nm SiO_2_/Si substrates. The thicknesses of the flakes are labeled. (**c**) The top view crystal structure of single-layer 1 T´-MoTe_2_. The zigzag metal chains, *a*-axis and *b*-axis are labeled. (**d**) The side view unit cells of 1 T´-MoTe_2_. (**e**) The high-resolution TEM image of few-layer 1 T´-MoTe_2_, where the direction of Mo-Mo chain is represented by a white double-headed arrow. (**f**) The selected electron diffraction image of 1 T´-MoTe_2_ flakes top-viewed from the [01-1] zone. (**g**,**h**) The representative 1 T´-MoTe_2_ and 2H-MoTe_2_ Raman spectra of 1-layer, 5-layer, and bulk.

The single-layer and few-layer 1 T´-MoTe_2_ were mechanically exfoliated from 1 T´-MoTe_2_ crystals (more details in Methods)^14,20^ onto a 285 nm SiO_2_/Si substrate (Fig. 1a–b). Their layer numbers were first identified by optical contrast and then confirmed by atomic force microscopy (AFM). In 1 T´-MoTe_2_, the formation of in-plane Mo-Mo bonds lead to a pseudo-hexagonal layer with zigzag metal chains (Fig. 1c). The layer stacking in 1 T´-MoTe_2_ is slightly tilted with *β* = 93.4° in a monoclinic fashion, resulting in the low symmetry (Fig. 1d). In order to obtain the details of the crystal structure, the exfoliated 1 T´-MoTe_2_ flakes are transferred to a micro-grid for high-resolution transmission electron microscopy (HR-TEM). The HR-TEM image displays a distinct one-dimensional chain (Fig. 1e), which corresponds to the crystalline direction of the *a*-axis. Furthermore, the selected area electron diffraction (SAED) pattern (Fig. 1f) confirms the clear monoclinic symmetry.

The representative Raman spectra of 1-layer, 5-layer, bulk MoTe_2_ in both the 1 T´ and 2 H (2D Semiconductors) phase are measured (Fig. 1g–h) using an excitation energy of 1.96 eV. The only modes observed in single-layer and bulk 2H-MoTe_2_ are the ${A}_{1}^{^{\prime} }$ (~172 cm^−1^) and *E*´(~233 cm^−1^) modes. In 5-layer 2H-MoTe_2_, apart from the corresponding ${A}_{1}^{^{\prime} }$ and *E*′ modes, an ${A}_{2}^{^{\prime\prime} }$ (~291 cm^−1^) mode, originates from the interlayer interactions under the 2D limit, can be detected. In addition, three Davydov components of the ${A}_{1}^{^{\prime} }$ modes in 5-layer, which originates from the in-phase and out-of-phase interlayer interactions^25,26^, are clearly observed. In contrast, several peaks at lower wave numbers are observed in 1 T´-MoTe_2_. For single-layer 1 T´-MoTe_2_, only three modes (~83, 165, 270 cm^−1^) can be observed. However, in 5-layer and bulk 1 T´-MoTe_2_, the number of detected modes increases. Given the same layer number, the number of detected modes in 1 T´-MoTe_2_ is greater than that in 2H-MoTe_2_, reflecting the lower crystal symmetry of the 1 T´ phase compared with the 2 H phase^31^.

### In-plane Anisotropic Raman Response by Rotating Sample

**Figure 2:**
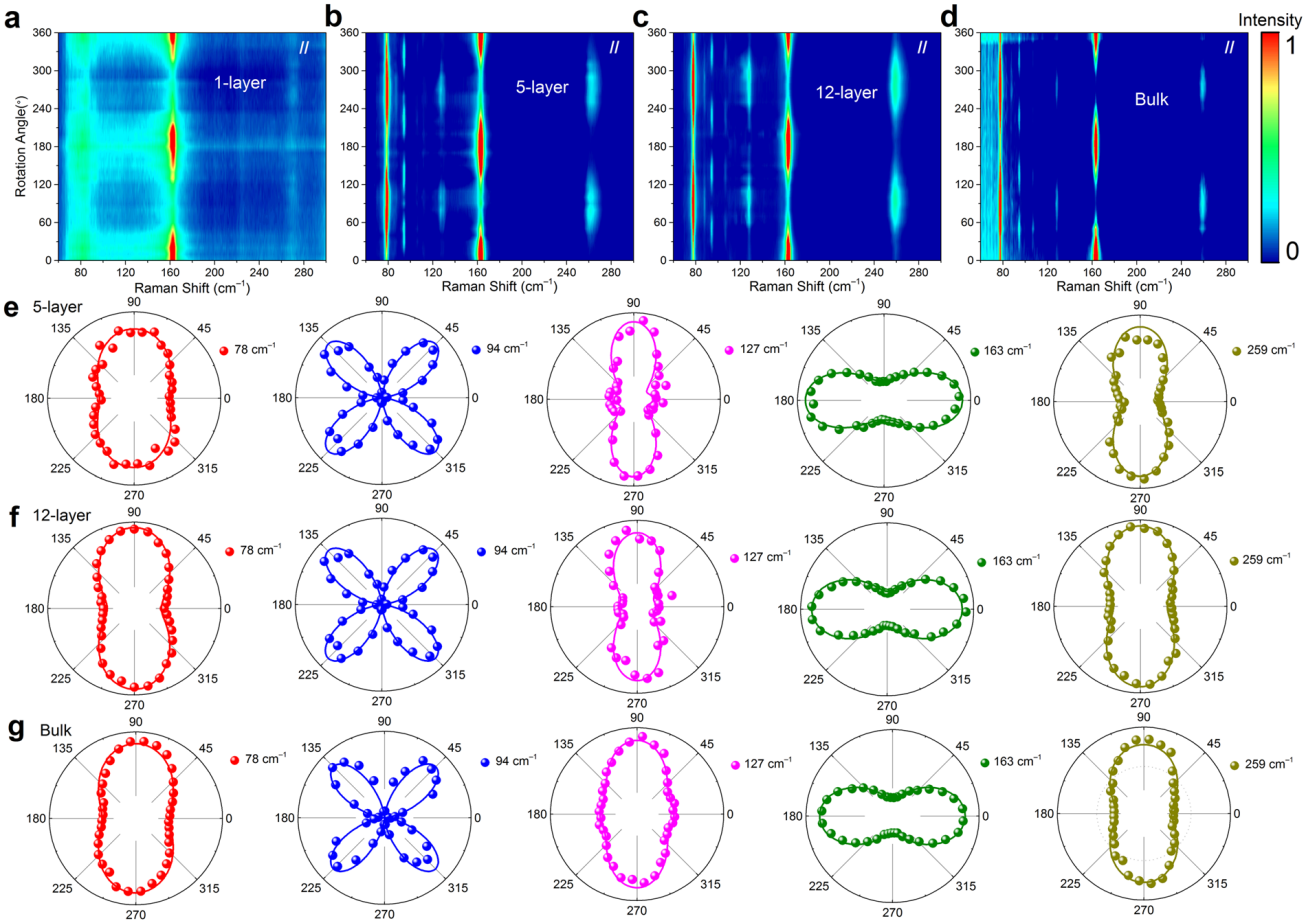
The sample rotation angle dependent Raman spectra of (**a**) 1-layer, (**b**) 5-layer, (**c**) 12-layer and (**d**) bulk 1 T´-MoTe_2_ in the parallel polarization configuration. The polar plots of the angle-dependent intensities of phonon modes in (**e**) 5-layer, (**f**) 12-layer and (**g**) bulk 1 T´-MoTe_2_. The initial angle is set when the crystalline *a*-axis is parallel to the incident polarization.

The angle-resolved Raman spectroscopy of 2D materials’ in-plane anisotropy can be carried out by rotating the sample or incident light polarization (more details in Methods)^11,30^. The rotating sample method was used to study the symmetry of these detected modes and identify crystalline orientation of the exfoliated flakes^10–12^. In this measurement, an analyzer was placed before the entrance of the spectrometer, allowing for the analysis of scattered Raman signal polarized parallel or perpendicular to the incident light polarization (parallel- or cross-polarization configurations, respectively). Raman spectra of 1-layer, 5-layer, 12-layer and bulk 1 T´-MoTe_2_ in the parallel-polarization configuration (Fig. 2a–d) exhibit strong rotation angle dependence. Only two modes could be clearly observed in single-layer 1 T´-MoTe_2_ in the range of 50–300 cm^−1^, due to the absence of interlayer coupling. Both modes show obvious 180° periodic variations with the sample rotation angle, yielding a two-lobed shape with two maximum intensity angles (Fig. 2a). The mode at 270 cm^−1^ is too weak to show a clear angle dependence. In 5-layer, 12-layer and bulk 1 T´-MoTe_2_, more Raman modes are detected (Fig. 2b–d). Some modes depict 180° periodic variations yielding two-lobed shapes in their polar plots (Fig. 2e–g), while others depict 90° periodic variations yielding four-lobed shapes in corresponding polar plots (Fig. 2e–g).

The angle dependences of five representative Raman modes (~78, 94, 127, 163 and 259 cm^−1^) in 5-layer, 12-layer and bulk 1 T´-MoTe_2_ are shown in Fig. 2e–g, respectively. The modes at ~78, 127, 163, and 259 cm^−1^ show obvious 180° periodic intensity variations, while the mode at ~94 cm^−1^ shows a 90° period. The main-axis of the two-lobed shapes at ~78, 127, and 259 cm^−1^ is perpendicular to the one-dimensional (1D) Mo-Mo chain (*a*-axis). However, the mode at ~163 cm^−1^ depicts a two-lobed shape with the main-axis parallel to the *a*-axis. In other words, the mode at ~163 cm^−1^ reaches its maximum intensity when the polarization of incident laser is along the well-defined Mo-Mo zigzag metal chain of the flakes. We have extensively measured more than twenty flakes with different thicknesses, and verified that the polarized angle dependence of ~163 cm^−1^ mode can be used to identify the crystalline orientation of 1 T´-MoTe_2_ flakes. The two-lobed modes can be further classified into two categories: the modes with maximum Raman intensity along or perpendicular to the 1D Mo-Mo chains under parallel polarization. These angle-dependent Raman intensities can be quantitatively understood based on the semi-classical analysis, which can be expressed as:^10^

1 $$I\propto {|{e}_{i}\cdot \tilde{R}\cdot {e}_{s}|}^{2}$$

where both the incident and scattered light polarization unitary vectors, *e*_*i*_ and *e*_*s*_, are (cos*θ*, sin*θ*, 0) in the parallel polarization configuration, *θ* is the angle between the incident laser polarization and the 1D Mo-Mo chain (*a*-axis), and $\tilde{R}$ is the Raman tensor for the Raman active modes of 1 T´-MoTe_2_. Bulk and odd numbered 1 T´-MoTe_2_ flakes belong to the space group *P*2_1_/*m* and point group ${{\rm{C}}}_{{\rm{2h}}}^{2}$, with 12 atoms and 6 *N* atoms in the unit cell, respectively. The irreducible representations for all possible phonon modes at the Brillion zone center *Г* point in bulk and odd numbered 1 T´-MoTe_2_ can be written as *Г*_bulk_ = 12*A*_g_ + 6*A*_u_ + 6*B*_g_ + 12*B*_u_ and *Г*_odd_ = 6*N A*_g_ + 3*N A*_u_ + 3*N B*_g_ + 6*N B*_u_, where *N* presents the number of layers. Even numbered 1 T´-MoTe_2_ flakes belong to the space group *Pm* and point group ${{\rm{C}}}_{{\rm{s}}}^{1}$. The irreducible representations of possible phonon modes at *Г* point in *N-* layer 1 T´-MoTe_2_ can be written as *Г*_even_ = 6*N* *A*′ + 12*N* *A*″. Among them, *A*_g_ and *B*_g_ are Raman active, *A*_u_ and *B*_u_ are infrared active, while *A*′ and *A*″ are both Raman and infrared active. It is worth noting that, in an absorptive material, the elements of the Raman tensors should be complex numbers, with real and imaginary parts^10^.

$$\tilde{{\rm{R}}}({A}_{{\rm{g}}}/A^{\prime} )=(\begin{array}{ccc}|a|{e}^{i{\varphi }_{a}} & 0 & |d|{e}^{i{\varphi }_{d}}\\ 0 & |b|{e}^{i{\varphi }_{b}} & 0\\ |d|{e}^{i{\varphi }_{d}} & 0 & |c|{e}^{i{\varphi }_{c}}\end{array})$$

$$\tilde{{\rm{R}}}({B}_{g}/A^{\prime\prime} )=(\begin{array}{ccc}0 & |e|{e}^{i{\varphi }_{e}} & 0\\ |e|{e}^{i{\varphi }_{e}} & 0 & |f|{e}^{i{\varphi }_{f}}\\ 0 & |f|{e}^{i{\varphi }_{f}} & 0\end{array})$$

Here, *ϕ* represents the phase of the corresponding tensor element^10,30^. A phonon mode can only be detected when ${|{e}_{i}\cdot \tilde{R}\cdot {e}_{s}|}^{2}\,$has non-zero value. Therefore, using parallel polarization, the above defined unitary vectors (*e*_i_ and *e*_s_), as well as the Raman tensors of the *A*_g_ (*A*′) and *B*_g_ (*A*″) modes, we can obtain their angular dependent Raman intensity expressions:

2 $${{{\rm{I}}}^{\parallel }}_{{A}_{g}({A}^{^{\prime} })}\propto {|a|}^{2}[{(|\frac{b}{a}|{\sin }^{2}\theta +{\cos }^{2}\theta \cos {\varphi }_{ba})}^{2}+{\cos }^{4}\theta {\sin }^{2}{\varphi }_{ba}]$$

3 $${{{\rm{I}}}^{\parallel }}_{{B}_{g}({A}^{^{\prime\prime} })}\propto {|e|}^{2}{\sin }^{2}2\theta $$

where 𝜙_ba_ is the phase difference 𝜙_b_-𝜙_a_. The experimental angle-dependent Raman intensities of the detected modes can be fitted by the equations (2) and (3) (Fig. 2e–f), yielding the corresponding 𝜙_ba_ and $|b/a|$ for *A*_g_ (*A*′) modes, summarized in Table 1. According to the above two equations, the modes with 180° periodic intensity variation (~78, 127, 163 and 259 cm^−1^) belong to *A*_g_ (*A*′) modes for odd- (even-) numbered 1 T´-MoTe_2_, while the ones with the 90° periodic intensity variation (94 and 115 cm^−1^) belong to *B*_g_ (*A*″) mode for odd- (even-) numbered 1 T´-MoTe_2_. It is worth noting that the mode at ~163 cm^−1^ is not the *B*_g_ mode as assigned in ref.^22^, because the intensity variation period is 180° (two-lobed shape), not 90° (four-lobed shape) in the parallel polarization configuration. According to equation (2), the *A*_g_ (*A*′) modes can also be classified into two types according to the Raman tensor element values |*a*|<|*b*| or |*a*|>|*b*|. Type I *A*_g_ (*A*′) modes (~78, 127 and 259 cm^−1^) with |*a*|<|*b*| result in a main-axis of the two-lobed shape perpendicular to the *a*-axis, while type II *A*_g_ (*A*′) modes (~163 cm^−1^) with |*a*|>|*b*| result in a main-axis of the two-lobed shape parallel to the *a*-axis. Therefore, the angle-dependent Raman spectroscopy in the parallel-polarized configuration provides an all-optical method for the identification of crystal orientation and can be used to determine the Raman modes’ symmetry in 1 T´-MoTe_2_.

**Table 1:** The fitted values of |*a*/*b*| and phase difference 𝜙_ba_ for the detected *A*_g_ (*A*′) modes in 5-layer, 12-layer and bulk 1 T´-MoTe_2_.

		78 cm^−1^	127 cm^−1^	163 cm^−1^	259 cm^−1^
5-layer	|*a*/*b*|	~1.40	~2.00	~0.50	~2.02
	cos*𝜙*_ba_	~1.00	~0.10	~0.60	~0.70
12-layer	|*a*/*b*|	~1.73	~2.01	~0.49	~1.74
	cos*𝜙*_ba_	~1.00	~0.30	~0.94	~0.90
bulk	|*a*/*b*|	~1.48	~1.41	~0.49	~1.58
	cos*𝜙*_ba_	~1.00	~0.60	~0.94	~0.98

### In-plane Anisotropic Raman Response by Rotating Incident Polarization

**Figure 3:**
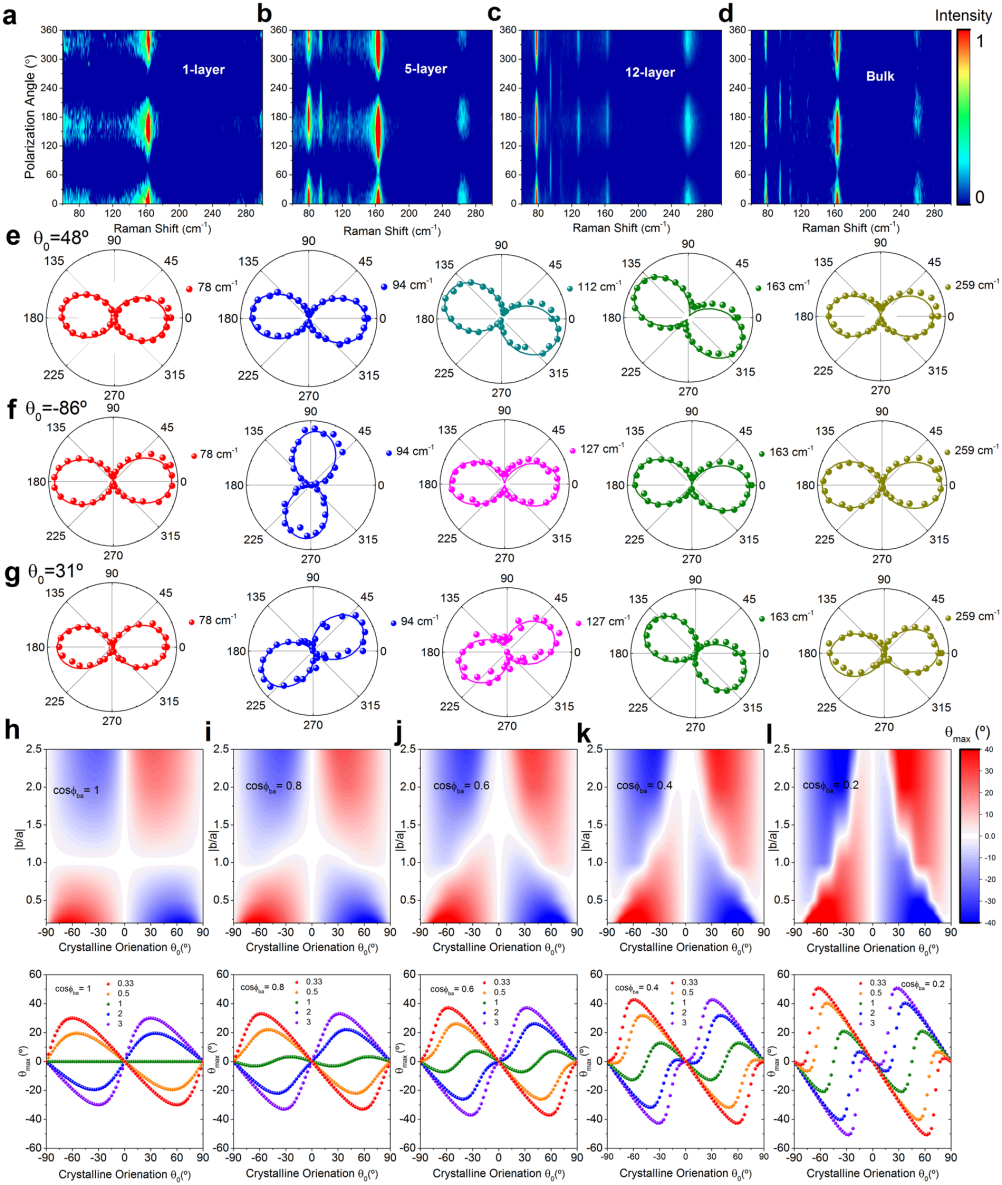
The rotation of the incident laser polarization dependent Raman spectra of (**a**) 1-layer, (**b**) 5-layer, (**c**) 12-layer and (**d**) bulk 1 T´-MoTe_2_, the initial incident polarization is parallel to the scattered polarization. Each spectrum has been normalized to its maximum intensity, respectively. The polar plots of the three detected mode types for (**e**) 5-layer, (**f**) 12-layer and (**g**) bulk 1 T´-MoTe_2_ samples. The calculated *θ*_max_ as a function of *θ*_0_ and |*b*/*a*|, where the value of cos 𝜙_ba_ is (**h**) 1, (**i**) 0.8, (**j**) 0.6, (**k**) 0.4, and (**l**) 0.2. The corresponding relation between *θ*_max_ and *θ*_0_ with certain |*b*/*a*| (0.33, 0.5, 1, 2 and 3) is plotted below each panel.

In order to further study the anisotropic Raman response of 1 T´-MoTe_2_, we carried out angle-dependent polarized Raman spectroscopy by rotating the incident laser polarization while fixing the sample and scattered light polarization. In the measurement, a half-wave plate was placed after the incident polarizer, allowing for the analysis of the scattered Raman signals with the incident laser polarization rotation angle of *θ*, while the half-wave plate was rotated by an angle of *θ*/2. Compared with the sample rotation method, the incident polarization rotation method is more convenient, as the half-wave plate rotation is programmable. At first, the incident laser polarization is set parallel to the scattered light polarization, and the angle between the scattered light polarization and the crystalline *a*-axis (obtained through the anisotropic Raman spectra in the parallel polarization configuration) is *θ*_0_. If the direction of the crystalline *a*-axis is clockwise compared with the scattered light polarization, the *θ*_*0*_ is positive, otherwise negative. The representative incident polarization dependent Raman spectra of 1-layer, 5-layer, 12-layer, and bulk MoTe_2_ were measured under the excitation energy of 1.96 eV (Fig. 3a–d). We can see that all the detected Raman modes (classified above) depict a two-lobed shape, but the main-axes are not simply parallel or perpendicular to the crystal *a*-axis (Fig. 3e–g). Considering the crystal symmetry and complex form of the Raman tensors, the above anisotropic results can be understood based on the semi-classical analysis, where the unit vectors of the incident and scattered light polarization are identical and can be expressed as (cos(*θ* + *θ*_0_), sin(*θ* + *θ*_0_), 0) and (cos*θ*_0_, sin*θ*_0_, 0). The angular dependent Raman intensity of *A*_g_ (*A*′) and *B*_g_ (*A*″) modes can be written as:

4 $${I}_{{A}_{g}(A\text{'})}\propto {|a|}^{2}{\cos }^{2}(\theta +{\theta }_{0}){\cos }^{2}{\theta }_{0}+{|b|}^{2}{\sin }^{2}(\theta +{\theta }_{0}){\sin }^{2}{\theta }_{0}+\frac{1}{2}|a||b|\sin [2(\theta +{\theta }_{0})]\sin \,2{\theta }_{0}\,\cos \,{\varphi }_{ba}$$

5 $${I}_{{B}_{g}({A}^{^{\prime\prime} })}\propto {|e|}^{2}{\sin }^{2}(\theta +2{\theta }_{0})$$

We can see that the intensity variation periods for all these modes are 180°. The main-axis orientation of the two-lobed shape is represented by the angle of the incident polarization with the maximum Raman intensity, *θ*_max_. From equation (4), *θ*_max_ of an *A*_g_ (*A*′) phonon mode should meet conditions where the first-order derivative is zero while the second-order derivative is negative:

6 $$({|b/a|}^{2}{\sin }^{2}{\theta }_{0}-{\cos }^{2}{\theta }_{0})\sin [2({\theta }_{0}+{\theta }_{{\rm{\max }}})]+|b/a|\cos [2({\theta }_{0}+{\theta }_{{\rm{\max }}})]\sin \,2{\theta }_{0}\,\cos \,{\varphi }_{ba}=0$$

and

7 $$({|b/a|}^{2}{\sin }^{2}{\theta }_{0}-{\cos }^{2}{\theta }_{0})\cos [2({\theta }_{0}+{\theta }_{{\rm{\max }}})]-|b/a|\sin [2({\theta }_{0}+{\theta }_{{\rm{\max }}})]\sin \,2{\theta }_{0}\,\cos \,{\varphi }_{ba} < 0$$

Similarly, *θ*_max_ of a *B*_g_ (*A*″) mode should meet:

8 $$2{\theta }_{0}+{\theta }_{{\rm{\max }}}=\pm 90^\circ $$

The values of *θ*_0_ and *θ*_max_ are set in the range of −90° to 90°. The *θ*_max_ is a function of *θ*_0_ and Raman tensor element ratio |*b*/*a*| (Fig. 3h–l). When the initial incident polarization is parallel to the crystal *a*-axis (*θ*_0_ = 0), the value of *θ*_max_ is zero. We can see that *θ*_max_ has an enhanced variation with *θ*_0_ when the Raman tensor element |*b*| deviates from |*a*|. This phenomenon can be clearly seen in Fig. 3h–l with |*b*/*a*| of 0.33, 0.5, 1, 2, and 3, respectively. For an absorptive material, the phase difference, 𝜙_b_-𝜙_a_, will change the dispersion of *θ*_max_. Figure 3h–l displays the evolution of the *θ*_max_ dispersion with an increased phase difference, represented by cos𝜙_ba_ values of 1, 0.8, 0.6, 0.4, and 0.2, respectively. Based on the above calculation, combined with the experimental extracted |*b*/*a*| values, the experimental *θ*_max_ of the *A*_g_ (*A*′) mode varies from about −40° to 40°, in good agreement with experimental observations. With the incident polarization rotation method, the above analysis demonstrates the main-axis orientation of the two-lobed *A*_g_ (*A*′) mode is not simply parallel or perpendicular to the crystal *a*-axis, but is a function of crystal orientation *θ*_0_, |*b*/*a*|, and the phase difference 𝜙_ba_.

### Excitation Energy Dependence

**Figure 4:**
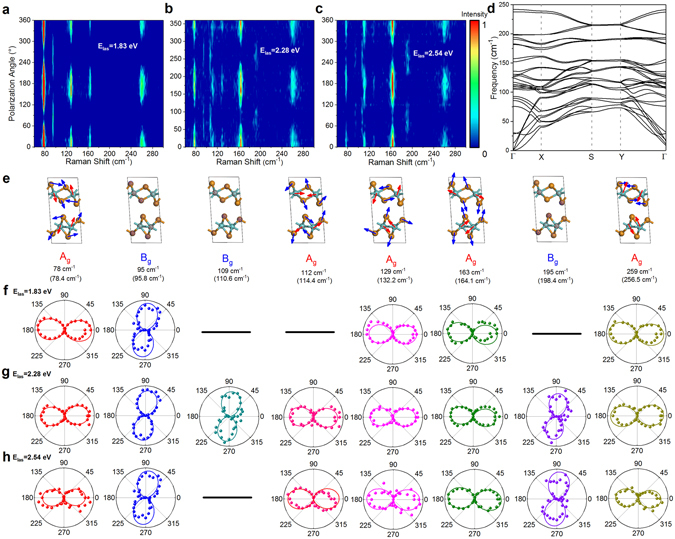
The incident laser polarization dependent Raman spectra of a 12-layer 1 T´-MoTe_2_ using an excitation energy of (**a**) 1.83, (**b**) 2.28 and (**c**) 2.54 eV. The initial incident polarization is parallel to the scattered polarization, and *θ*_0_ = −86°. Each spectrum has been normalized to its maximum intensity, respectively. (**d**) Calculated phonon dispersion curves of 1 T´-MoTe_2_ bulk along the Г-X-S-Y-Г direction in the Brillouin zone. (**e**) The calculated atomic displacements for the lattice vibrations of the ten detected modes in 1 T´-MoTe_2_, together with their corresponding irreducible representations. The theoretical frequency is given below in each panel. The atomic displacements of Mo (Te) atoms are shown in red (blue) arrows. The Raman intensities were given as polar plots of the detected modes with an excitation energy of (**f**) 1.83, (**g**) 2.28 and (**h**) 2.54 eV.

The number of detected modes in few-layer 1 T´-MoTe_2_ is limited compared with that in Td-WTe_2_^12^. In order to detect more modes and further study the Raman response of 1 T´-MoTe_2_ on polarization, phonon and photon energies, we carried out the anisotropic Raman measurements with three additional excitation energies (1.83, 2.28, and 2.54 eV) via rotating the incident polarization. The 12-layer 1 T´-MoTe_2_ flake with *θ*_0_ = −86° was chosen as the representative sample, due to the considerable number and intensity of the detectable modes as well as the easily identifiable mode symmetry. Figure 4a–c shows the polarization-dependent Raman spectra of 12-layer 1 T´-MoTe_2_ under these three excitation energies, respectively. A total of 8 modes can be resolved with an excitation energy of 2.28 eV, while some modes are missing with an excitation energy of 1.83 and 2.54 eV. We calculated the phonon dispersion curve of bulk 1 T´-MoTe_2_ (Fig. 4d) using density functional theory (DFT) and found all 36 normal modes at the Brillouin zone center *Г* point are non-degenerate in bulk 1 T´-MoTe_2_, which differs from that of 2H-type TMDCs with higher symmetry (the modes vibrating parallel to the 2D plane are double degenerate). The measured Raman frequencies and identified mode symmetries agree well with the calculated phonon modes from DFT (Fig. 4e). The atomic displacements for the lattice vibration of detected modes and their polarization-dependent intensities using excitation energies of 1.83, 2.28, and 2.54 eV are shown in Fig. 4e–h, respectively. Based on equations (6–8), for a *θ*_0_ of −86° (closer to −90°), the main-axis orientation *θ*_max_ of *A*_g_ (*A*′) mode is closer to zero, and that of *B*_g_ (*A*″) mode is closer to ± 90°. Then the modes at 78, 112, 127, 163 and 259 cm^−1^ are *A*_g_ (*A*′) modes, while the ones at 95, 109, and 195 cm^−1^ are *B*_g_ (*A*″) modes. The measured Raman frequencies and mode symmetries agree well with those of the DFT calculated phonon modes (Fig. 4e–h and supplementary Table S1). The atoms of the *B*_g_ (*A*″) modes vibrate along the Mo-Mo zigzag chain, while the atoms of the *A*_g_ (*A*′) modes vibrate within the mirror plane perpendicular to the chain. We conclude that 2.28 eV is an appropriate excitation energy to detect more Raman modes in few-layer 1 T´-MoTe_2_, and the symmetry of detected modes can be easily identified in the incident polarization rotation method.

### Full Quantum Model of Anisotropic Raman Response

**Figure 5:**
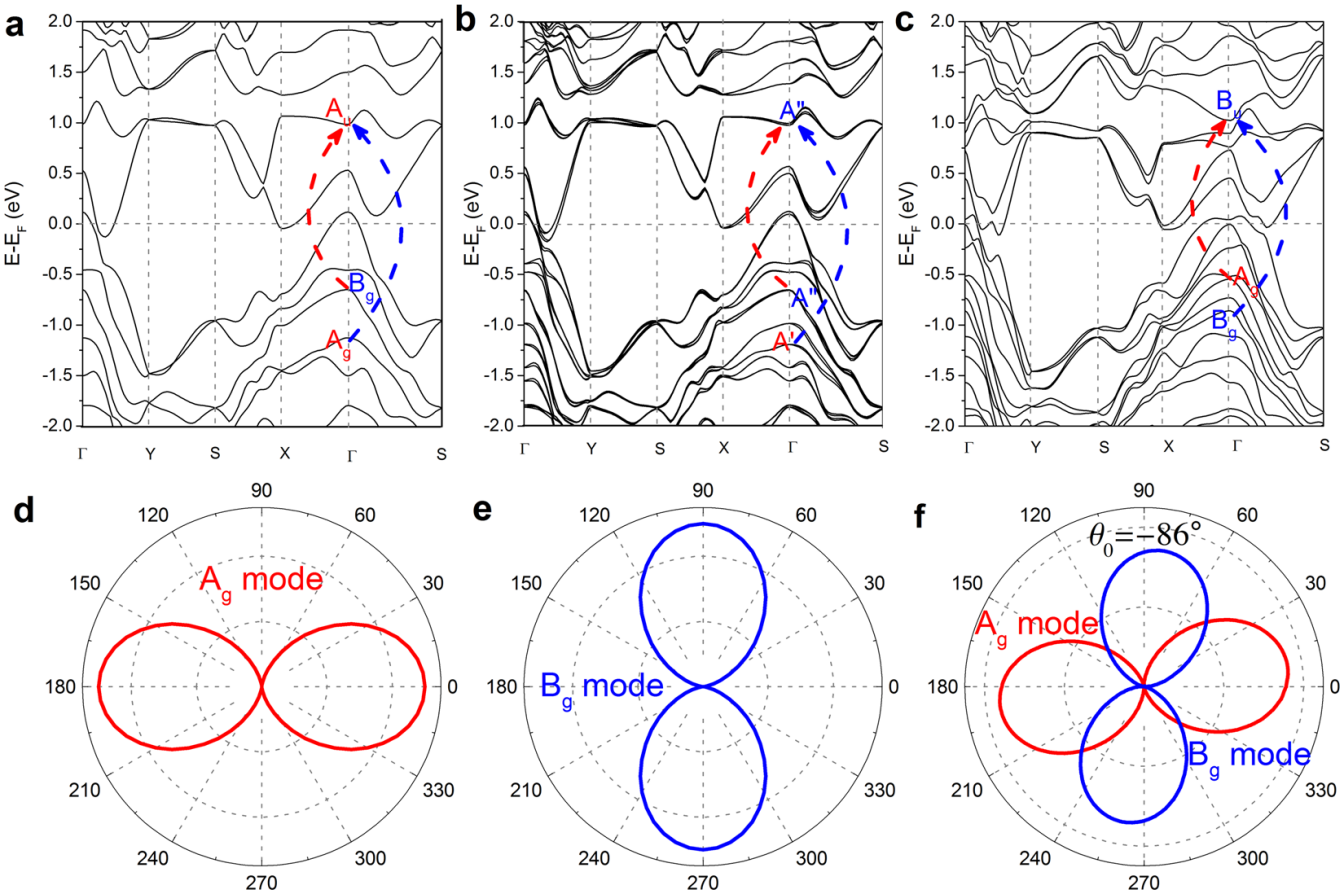
The density functional calculated band structure of (**a**) single-layer, (**b**) bilayer and (**c**) bulk 1 T´-MoTe_2_, the band symmetries in the Brillouin zone center *Г* point are labeled, and *A*_g_ (*A*′, *A*_u_) and *B*_g_ (*A*″, *B*_u_) bands are indicated by red and blue labels, respectively. Calculated polarization dependence of the optical transition probability (**d**) with the maximum *a*-axis polarization (*A*_g_ mode) and (**e**) the maximum *b*-axis polarization (*B*_g_ mode). The 0° (90°) corresponds to the *a*-axis (*b*-axis) of the 1 T´-MoTe_2_ sample. (**f**) Calculated polarization-dependence of the *A*_g_ and *B*_g_ phonon mode intensities, from the incident polarization rotation method.

To further explore the physical origin of the anisotropic Raman response, we employed the full quantum model based on DFT calculation and quantum perturbation theory^28^. The Raman scattering response contains three processes: incident photon absorption, the creation/destruction of the phonon, and scattered photon emission. The incident photon absorption and scattered photon emission processes are related to the electron-photon interactions, while the creation/destruction of the phonon is related to the electron-phonon interaction. The Raman intensity with incident laser photon energy (*E*_las_) can be obtained by incorporating two electron-photon matrix elements and one electron-phonon matrix element as follows:

9 $${\rm{I}}({E}_{las})={|\sum _{i,m,n}\frac{\langle f|{H}_{e-p}|n\rangle \langle n|{H}_{e-ph}|m\rangle \langle m|{H}_{e-p}|i\rangle }{({E}_{las}-{E}_{m}-i{\Gamma }_{m})({E}_{las}-{E}_{n}-i{\Gamma }_{n})}|}^{2}$$

where 〈*m*|*H*_*e−p*_|*i*〉 and 〈*f*|*H*_*e−p*_|*n*〉 represent the electron-photon interaction processes, while 〈*n*|*H*_*e−ph*_|*m* 〉represents the electron-phonon interaction process. The terms |*i*〉, |*f* 〉, |*m*〉, and |*n*〉 are the initial, final, and two intermediate states, respectively. *E*_m_, and *E*_n_ are the energies of the two intermediate states |*m*〉 and |*n*〉. *Г*_m_ and *Г*_n_ are the damping constants related to the finite lifetimes of the two intermediate states. The electron-photon interaction contains the transition of the excited electron from the valence band to conduction band, protected by the optical selection rule. In order to explain the observed anisotropies of the Raman response in 1 T´-MoTe_2_, we performed electron-photon interaction calculations. We calculated the representative band structures of 1-layer, 2-layer, and bulk 1 T´-MoTe_2_. The symmetry of the eigen-function for each energy band at the *Г* point was determined using first-principles calculations (Fig. 5a–c). If we neglect the polarization dependence of the electron-phonon matrix element 〈*n*|*H*_*e−ph*_|*m*〉, the polarization dependence of the Raman intensity can be described by the product of two electron-photon matrix elements, 〈*f*|*H*_*e−p*_|*n*〉 and 〈*m*|*H*_*e−p*_|*i*〉. In Raman scattering, *H*_*e−ph*_ selects the symmetry of |*m*〉 and |*n*〉, while the initial |*i*〉 and final |*f*〉 states are the same. For the *A*_g_ (*A*′) mode, the |*m*〉 and |*n*〉 states have the same symmetry, and the two electron-photon interaction matrix elements in equation (9) have the same polarization dependence (180° period in the parallel-polarization by rotating the sample). On the other hand, the *B*_g_ (*A*″) mode’s |*m*〉 and |*n*〉 states have different symmetries, and the two matrix elements have the opposite polarization dependence that gives the 90° period. These are the optical transition selection rules which can be explained using group theory. The detailed selection rules for *A*_g_ (*A*′) and *B*_g_ (*A*″) Raman modes are summarized in supplementary Tables S2 and S3, respectively. Depending on the incident and scattered polarized light, we can excite *A*_g_ (*A*′) phonon modes for *aa* and *bb* polarizations, and excite *B*_g_ (*A*″) phonon modes for *ab* and *ba* polarizations. Here, we use the notation *ab* polarization to describe the Raman process with incident polarized light along *a*-axis and scattered polarized light along *b*-axis of the crystals. The other polarization conditions *aa*, *ba*, and *bb* are defined accordingly.

For detailed analysis, we take the* ﻿Г* point in﻿ single-layer 1 T´-MoTe_2_ as an example. For *E*_*las*_ ≈ 1.8 eV, the transition between the *B*_g_ valence band and the *A*_u_ conduction band occurs when incident light polarization is along the *a*-axis. According to the symmetry selection rule, the polar plot of the transition probability with an incident light polarization rotation angle, *θ*, shows a two-lobed shape (Fig. 5d), where the maximum absorption occurs when incident polarized light is long the *a*-axis. In the incident light polarization rotation method, since the scattered polarization is fixed, the photon emission process has no influence on the Raman intensity’s polarization dependence^28^. When the scattered light polarization is along the *a*-axis (*θ*_0_ = 0°), this transition dispersion coincides with the calculated polarization-dependence of the *A*_g_ phonon mode’s intensity. For *E*_*las*_ ≈ 2.2 eV, the transition between the *A*_g_ valence band and the *A*_u_ conduction band occurs when the incident polarized light is perpendicular to the crystalline *a*-axis. The polar plot of the transition probability coincides with the polarization-dependence of the *B*_g_ phonon mode (Fig. 5e), showing a two-lobed shape with maximum values when the incident light polarization is perpendicular to the crystalline *a*-axis. Similarly, the transition between the *A*″ (*A*_g_) valence band and the *A*″ (*B*_u_) conduction band occur when incident polarized light polarization is along the *a*-axis for bilayer (bulk) 1 T´-MoTe_2_, while the transitions between the *A*′ (*B*_g_) valence band and the *A*″ (*B*_u_) conduction band occur when incident polarized light is perpendicular to the *a*-axis for bilayer (bulk) 1 T´-MoTe_2_. For the case that the scattering light polarization is not parallel to the crystalline *a*-axis (*θ*_0_ ≠ 0°), the representative polarization-dependence of the *A*_g_ and *B*_g_ phonon modes with *θ*_0_ = −86° are calculated (Fig. 5f). These analytical results agree with our anisotropic experimental observations, but do not perfectly coincide with all the data points. For example, in the parallel polarization configuration, the formation of secondary maxima and non-zero minimum intensity in the *A*_g_ phonon modes cannot be completely explained by the anisotropic electron-photon interaction, revealing that the anisotropic electron-phonon interaction also contributes to its anisotropic Raman response.

## Conclusions

We systematically studied the anisotropic Raman response of monoclinic 1 T´-MoTe_2_ from single layer to bulk using two different measurement methods. Through sample rotation method, the symmetries of the detected modes and the crystal orientation can be identified due to the different intensity variation periods. The thickness and excitation wavelength dependences of the anisotropic Raman response of 1 T´-MoTe_2_ are carried out via rotating the incident polarization, while the sample orientation and scattered light polarization are fixed. Under this method, the polar plots of the intensities for all detected modes exhibit a two-lobed shape, while the main-axis orientations for different symmetric modes are different. Their polarization-dependent intensities roughly agree with the semi-classical model based on the Placzek approximation. Using the full quantum model based on the density functional and quantum perturbation theories, we demonstrate that the anisotropy of the Raman modes is influenced by both the anisotropic electron-photon interaction as well as the anisotropic electron-phonon interaction. Our research not only presents the in-plane anisotropic intensities of Raman modes in monoclinic 2D 1 T´-MoTe_2_, but also reveals the physical origin of the anisotropic Raman response, which is a crucial step to enable potential applications of 1 T´-MoTe_2_ in novel electronic and optoelectronic devices where the anisotropic properties might be utilized.

## Methods

### Single-crystalline 1 T´-MoTe_2_ growth

1 T´-MoTe_2_ single crystals were grown via a chemical vapor transport (CVT) technique. Stoichiometric purified Mo (Sigma Aldrich 99.99%) and Te (Alfa Aesar 99.99%) powders were ground together and loaded into a quartz tube with a small amount of I_2_ as the transport agent. The tube was sealed under vacuum, then placed and heated in a two-zone furnace. The hot and cold zones were kept at 1000 °C and 850 °C, respectively. After 10 days of growth, the 1 T´-MoTe_2_ single crystals were obtained in the cold zone.

### Raman measurements

The Raman measurements were carried out using a commercial micro-Raman system (Horiba Jobin Yvon HR800) under the backscattering geometry. A 100× object lens (NA = 0.85), and a 1800 grooves/mm grating were used in our measurements. The exposure time was 100 s. The laser power illuminated on the sample was below 400 μW to avoid the sample damage. All the measurements were carried out at room temperature.

### Density functional calculations

The DFT calculations were carried out with the Vienna *ab-initio* Simulation Package (VASP)^32^ based on density functional theory. The Perdew-Burke-Ernzerhof (PBE) exchange-correlation functional^33^ along with the projector-augmented wave (PAW) potentials was employed for the self-consistent total energy calculations and geometry optimization. The kinetic energy cutoff for the plane wave basis set was chosen to be 500 eV for all calculation. The Brillioun zones were sampled using 7 × 14 × 3 (for bulk 1 T´-MoTe_2_) and 7 × 14 × 1 (for 1L-3L 1 T´-MoTe_2_) Monkhorst-Pack k-point grid. Atomic positions were relaxed until the energy differences were converged within 10^−5^ eV and the maximum Hellmann-Feynman force on any atoms was below 0.01 eV/Å. We used a periodic supercell to simulate a 2D sheet, including a vacuum of 15 Å to separate the adjacent periodic images of the sheet. The spin-orbit interaction (SOC) was included in the calculation of electronic structure of bulk and few-layer 1 T´-MoTe_2_. The van der Waals (vdW) interactions were considered at the vdW-DF^34,35^ level for geometry optimization, which was found to be more accurate in describing the structural properties of layered materials.

## Electronic supplementary material

Supporting information for Anomalous in-plane anisotropic Raman response of monoclinic semimetal 1T´-MoTe2
