# Author Correction: Anomalous in-plane anisotropic Raman response of monoclinic semimetal 1 T′-MoTe_2_

**DOI:** 10.1038/s41598-017-17245-w

**Published:** 2017-12-15

**Authors:** Qingjun Song, Haifeng Wang, Xingchen Pan, Xiaolong Xu, Yilun Wang, Yanping Li, Fengqi Song, Xiangang Wan, Yu Ye, Lun Dai

**Affiliations:** 1 State Key Lab for Mesoscopic Physics and School of Physics, Peking University, Beijing, 100871 China; 2 Collaborative Innovation Center of Quantum Matter, Beijing, 100871 China; 3 National Laboratory of Solid State Microstructures, College of Physics, Nanjing University, Nanjing, 210093 China; 4 Collaborative Innovation Center of Advanced Microstructures, Nanjing University, Nanjing, 210093 China

*Scientific Reports* **7**:1758; doi:10.1038/s41598-017-01874-2; Article published online 11 May 2017

This Article contains typographical errors in Table 1. The correct Table 1 appears below as Table 1. As a result, the Table title,

**Table 1:** .

		78 cm^−1^	127 cm^−1^	163 cm^−1^	259 cm^−1^
5-layer	|*b*/*a*|	~1.40	~2.00	~0.50	~2.02
	cos *ϕ*_ba_	~1.00	~0.10	~0.60	~0.70
12-layer	|*b*/*a*|	~1.73	~2.01	~0.49	~1.74
	cos *ϕ*_ba_	~1.00	~0.30	~0.94	~0.90
bulk	|*b*/*a*|	~1.48	~1.41	~0.49	~1.58
	cos *ϕ*_ba_	~1.00	~0.60	~0.94	~0.98

“The fitted values of |*a*/*b*| and phase difference 𝜙_ba_ for the detected *A*_g_ (*A*′) modes in 5-layer, 12-layer and bulk 1 T′-MoTe_2_”.

should read:

“The fitted values of |*b*/*a*| and phase difference 𝜙_ba_ for the detected *A*_g_ (*A*′) modes in 5-layer, 12-layer and bulk 1 T′-MoTe_2_”.

