# Supplementary material for: Anomalous in-plane anisotropic Raman response of monoclinic semimetal 1 T´-MoTe2
Source: Sci Rep. 2017 May 11;7:1758. doi: 10.1038/s41598-017-01874-2 (PMC5431984; doi:10.1038/s41598-017-01874-2)
Supplement: Supplementary file 1 — Supporting information for Anomalous in-plane anisotropic Raman response of monoclinic semimetal 1T´-MoTe2 [file 41598_2017_1874_MOESM1_ESM.doc]

Supporting information for

**Anomalous in-plane anisotropic Raman response of monoclinic semimetal 1T´-MoTe_2_**

Qingjun Song,^1, 2^ Haifeng Wang,^3, 4^ Xingchen Pan,^3, 4^ Xiaolong Xu,^1, 2^ Yilun Wang,^1, 2^ Yanping Li,^1^ Fengqi Song,^3, 4^ Xiangang Wan,^3, 4, *^ Yu Ye ^1, 2, *^ and Lun Dai ^1, 2, *^

^1^ State Key Lab for Mesoscopic Physics and School of Physics, Peking University, Beijing 100871, China.

^2^ Collaborative Innovation Center of Quantum Matter, Beijing 100871, China.

^3^ National Laboratory of Solid State Microstructures, College of Physics, Nanjing University, Nanjing 210093, China.

^4^ Collaborative Innovation Center of Advanced Microstructures, Nanjing University, Nanjing 210093, China.

* Correspondence and requests for materials should be addressed to Xiangang Wan, Yu Ye & Lun Dai. (Email: xgwan@nju.edu.cn, ye_yu@pku.edu.cn, lundai@pku.edu.cn).

**Table S1.** The irreducible representations, activities, calculated frequencies for 36 phonon modes in bulk 1T´-MoTe_2_ and the experimental frequencies for the detected modes. Here, R and IR represent Raman active and infrared active, respectively.

| Number | Irreducible representation | Activity | DFT Calculated frequencies  (cm^−1^) | Experimental frequencies  (cm^−1^) |
| --- | --- | --- | --- | --- |
| 1 | *B*_u_ | IR | −4.9 |  |
| 2 | *B*_u_ | IR | −2.3 |  |
| 3 | *A*_u_ | IR | 11.8 |  |
| 4 | *B*_u_ | IR | 15.8 |  |
| 5 | *B*_u_ | IR | 37.7 |  |
| 6 | *A*_u_ | IR | 37.8 |  |
| 7 | *A*_g_ | R | 78.4 | 78 |
| 8 | *A*_g_ | R | 90.7 | 90 |
| 9 | *B*_g_ | R | 90.7 |  |
| 10 | *B*_g_ | R | 95.8 | 95 |
| 11 | *B*_g_ | R | 110.6 | 109 |
| 12 | *B*_g_ | R | 113.3 |  |
| 13 | *A*_g_ | R | 114.4 | 112 |
| 14 | *A*_u_ | IR | 114.4 |  |
| 15 | *A*_g_ | R | 118.3 |  |
| 16 | *A*_u_ | IR | 118.3 |  |
| 17 | *B*_u_ | IR | 121.0 |  |
| 18 | *B*_u_ | IR | 129.4 |  |
| 19 | *A*_g_ | R | 132.2 | 129 |
| 20 | *A*_g_ | R | 134.0 |  |
| 21 | *B*_u_ | IR | 134.5 |  |
| 22 | *B*_u_ | IR | 141.0 |  |
| 23 | *A*_g_ | R | 156.0 |  |
| 24 | *A*_g_ | R | 164.1 | 163 |
| 25 | *A*_g_ | R | 189.2 |  |
| 26 | *A*_u_ | IR | 190.3 |  |
| 27 | *B*_g_ | R | 198.4 | 195 |
| 28 | *B*_g_ | R | 204.4 |  |
| 29 | *B*_u_ | IR | 207.2 |  |
| 30 | *B*_u_ | IR | 207.8 |  |
| 31 | *A*_g_ | R | 244.4 |  |
| 32 | *A*_g_ | R | 250.4 |  |
| 33 | *A*_g_ | R | 268.6 | 261 |
| 34 | *A*_g_ | R | 269.9 |  |
| 35 | *B*_u_ | IR | 275.2 |  |
| 36 | *B*_u_ | IR | 276.8 |  |

**Table S2.** Selection rules for intermediate states
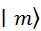
 and
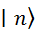
 for a given initial state, and for polarization vector *aa* or *bb* which both correspond to the excitation of *A*_g_ (*A*´) phonon mode. These selection rules correspond to the following product of matrix elements:
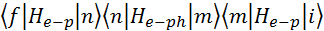
, with *i* = *f*

Odd-layer and bulk 1T´-MoTe_2_

| *aa* | *bb* |
| --- | --- |
| 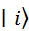 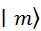 = 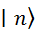 | 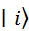 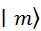 = 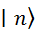 |
| A_g_ B_u_  B_g_ A_u_  A_u_ B_g_  B_u_ A_g_ | A_g_ A_u_  B_g_ B_u_  A_u_ A_g_  B_u_ B_g_ |

Even-layer 1T´-MoTe_2_

| *aa* | *bb* |
| --- | --- |
| 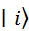 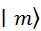 = 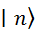 | 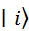 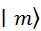 = 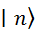 |
| A′ A′  A″ A″ | A′ A″  A″ A′ |

**Table S3.** Selection rules for intermediate states
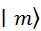
 and
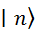
for a given initial state *i*, and polarization vector *ab* or *ba* which both correspond to the excitation of *B*_g_ (*A*″) phonon from single-layer to bulk 1T´-MoTe_2_, These selection rules correspond to the following product of matrix elements.
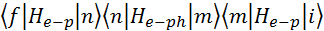
, with *f* = *i*

Odd-layer and bulk 1T´-MoTe_2_

| *ab* | *ba* |
| --- | --- |
| 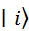 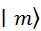 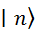 | 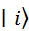 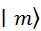 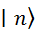 |
| A_g_ B_u_ A_u_  B_g_ A_u_ B_u_  A_u_ B_g_ A_g_  B_u_ A_g_ B_g_ | A_g_ A_u_ B_u_  B_g_ B_u_ A_u_  A_u_ A_g_ B_g_  B_u_ B_g_ A_g_ |

Even-layer 1T´-MoTe_2_

| *ab* | *ba* |
| --- | --- |
| 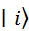 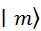 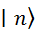 | 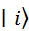 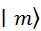 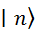 |
| A′ A′ A″  A″ A″ A′ | A′ A″ A′  A″ A′ A″ |
